# Research on Cavitation Fault Diagnosis of Axial Piston Pumps Based on Rough Set Attribute Weighted Convolutional Neural Networks

**DOI:** 10.3390/s25216769

**Published:** 2025-11-05

**Authors:** Min Liu, Zhiqi Liu, Jinyuan Cui, Yigang Kong, Zhipeng Ma, Wenwen Jiang, Le Ma

**Affiliations:** 1 College of Mechanical Engineering, Taiyuan University of Science and Technology, Taiyuan 030024, China; liumin1@stu.tyust.edu.cn (M.L.); cuijinyuan0008@link.tyut.edu.cn (J.C.); yigangkong@tyust.edu.cn (Y.K.); b202412110007@stu.tyust.edu.cn (Z.M.); jiangwenwen@tyust.edu.cn (W.J.); s202312110030@stu.tyust.edu.cn (L.M.); 2 Key Laboratory of Advanced Transducers and Intelligent Control System of Ministry of Education, Taiyuan University of Technology, Taiyuan 030024, China

**Keywords:** piston pump, cavitation fault, rough set theory, weight, interpretability

## Abstract

Cavitation phenomenon in piston pumps not only causes vibration and noise but also leads to component damage. Conventional diagnostic methods suffer from low accuracy, while deep learning approaches lack interpretability. To address these limitations, this paper proposes an intelligent fault diagnosis method based on the rough set Attribute Weighted Convolutional Neural Network (RSAW-CNN). First, based on cavitation mechanisms and the mathematical model, the computational fluid dynamics model of the piston pump is established to simulate the failure condition. Subsequently, employing rough set theory, an original fault decision table is constructed, discretized, and subjected to attribute reduction. A weight matrix is generated according to the importance of each data channel in the classification decision and embedded into the input layer of the Convolutional Neural Network (CNN) to enhance the influence of key features. Decision rules are also extracted to provide interpretable decision support for fault diagnosis. Experimental results demonstrate that the proposed RSAW-CNN method achieves an average diagnostic accuracy of over 99.2%. Compared to the backpropagation neural network, residual neural network, CNN, and the CNN with squeeze-and-excitation networks, its average accuracy has improved by 15.87%, 10.83%, 7.48%, and 5.40%. The proposed method not only exhibits high diagnostic accuracy but also offers strong interpretability and reliability.

## 1. Introduction

Axial piston pumps serve as the power source of high-end equipment hydraulic systems, delivering continuous and stable hydraulic power to actuators [1]. Once a malfunction in a piston pump can compromise the entire hydraulic system, leading to degraded equipment performance, reduced operational efficiency, and even complete system failure [2]. Among these failures, cavitation represents a particularly common and detrimental type of failure. Prolonged cavitation significantly shortens the service life of piston pump components, potentially causing critical failures such as damage to the valve plate or cracking of the cylinder block [3,4]. Such failures not only incur substantial repair costs but also result in production delays or safety incidents due to unexpected equipment downtime [5,6]. This underscores the significant value of cavitation fault diagnosis research.

Since cavitation occurs internally within the pump, technicians cannot directly observe and make precise judgments about its occurrence based on visual inspection alone [7]. Furthermore, cavitation faults often manifest as weak characteristic signals with low distinguishability, presenting considerable challenges for accurate diagnosis [8]. Traditional diagnostic approaches, including visual observation, pressure/flow signal analysis, and vibration signal analysis, have inherent limitations [9,10]. High-speed camera visualization allows direct observation of cavitation phenomena [11] but lacks sensitivity in capturing micro-cavitation and intermittent cavitation [12]. Quantitative assessment of cavitation through pressure and flow signal analysis is possible, yet it frequently misdiagnoses early subtle changes [13,14]. In contrast, vibration signal analysis is highly sensitive to the high-frequency shock responses induced by cavitation [15]. Nevertheless, the complexity of vibration sources necessitates specialized signal processing techniques to effectively distinguish cavitation from other mechanical faults [16,17]. Therefore, integrating multiple diagnostic methods is a promising approach to enhance both accuracy and reliability.

The advancement of artificial intelligence has spurred the adoption of intelligent diagnostic methods [18]. Machine learning techniques (e.g., decision trees, support vector machines, neural networks) [19] and multi-channel CNN models [20] have shown promise but suffer from data redundancy and poor interpretability [21]. Composite approaches integrating data preprocessing have addressed these issues to some extent: wavelet analysis combined with rough set rule generation and fuzzy classification [22], improved CNN variants [23,24], and transient flow measurement with enhanced transfer learning [25] have optimized feature extraction and reduced redundancy [26]. Collectively, these composite methods reduce data redundancy through preprocessing, and optimize feature extraction, leading to improved model performance and generalization capabilities [27,28].

In summary, composite fault diagnosis methods not only leverage the synergistic advantages of multiple techniques to improve diagnostic accuracy but also provide more intuitive insights, thereby strengthening operators trust and understanding of equipment condition [29,30]. Although deep learning models such as convolutional neural network (CNN) have demonstrated great potential in the field of fault diagnosis for axial piston pumps, they still face challenges, including low diagnostic efficiency, poor interpretability, and insufficient credibility. Deep learning models fail to identify the key input features causing faults, leading to a lack of targeting in subsequent maintenance work. Their “black-box” nature prevents users from directly participating in the decision-making process, greatly reducing user trust and severely limiting their practical application value. To address these issues, there is an urgent practical demand for an intelligent diagnostic model with both high accuracy and strong interpretability. This paper proposes an intelligent fault diagnosis method based on a rough set attribute-weighted convolutional neural network (RSAW-CNN), enhancing the accuracy and interpretability of fault diagnosis. It provides a novel approach to improving the intelligence and reliability of axial piston pumps. Our contributions are as follows:

(1) Proposal of a novel Rough Set-based Attribute Weighted Convolutional Neural Network (RSAW-CNN) model for fault diagnosis. This model achieves a deep integration of the knowledge discovery capability inherent in rough set theory and the deep learning power of a one-dimensional convolutional neural network (1DCNN). Guided by the feature importance derived from rough set theory, the CNN learning process is enhanced, effectively improving both model convergence speed and diagnostic accuracy. Furthermore, the decision rules obtained through rough set theory augment the model’s fault diagnosis efficiency and interpretability.
(2) Introduction of an innovative weighting mechanism for the 1DCNN. Based on rough set theory, an attribute weight matrix is generated to quantify the importance of each input channel. This matrix is embedded into the input layer of the CNN, thereby incorporating derived prior knowledge at the initial stage of model learning. Guiding the CNN learning based on input feature importance directs the network focus towards identified critical attributes, enhancing the efficiency and targeted nature of feature learning.
(3) Achievement of dual interpretability concerning both the diagnostic process and the underlying fault mechanism. Based on the physical mechanism and mathematical model of cavitation, an original fault decision table for cavitation faults is constructed. Following rough set-based discretization and attribute reduction, rules for cavitation faults are derived to serve as the decision basis. When physical parameters, such as piston pump outlet pressure and outlet flow rate, fall within specific threshold ranges, concrete cavitation fault states are deduced. This provides engineers with valuable, interpretable analysis concerning the fault mechanism, thereby enhancing the reliability and trustworthiness of the diagnostic system.

The remainder of this paper is structured as follows: Section 2 introduces the cavitation failure mechanisms and mathematical models. Section 3 describes the computational fluid dynamics simulation analysis of the piston pump. Section 4 details the proposed methodology, incorporating rough set theory and convolutional neural networks. Section 5 presents the RSAW-CNN fault diagnosis model and discusses the diagnostic results. Finally, Section 6 provides the conclusion of the study.

## 2. Cavitation Failure Mechanisms and Mathematical Models

### 2.1. Cavitation Failure Mechanisms

**Figure 1:**
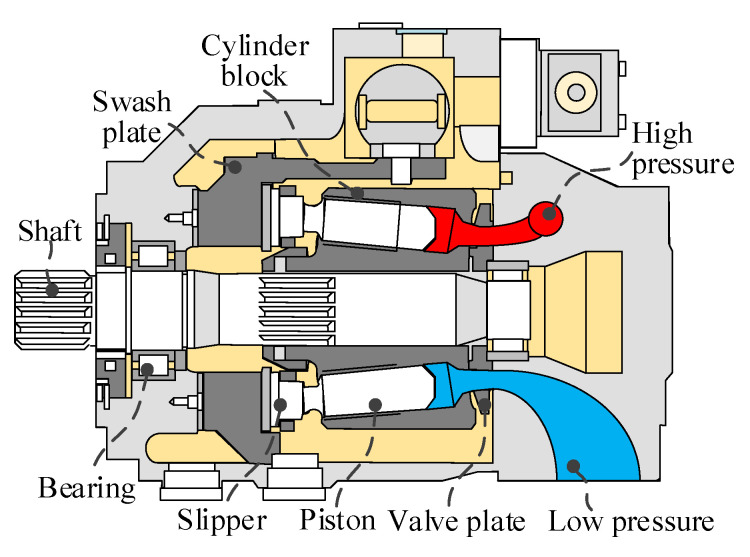
Schematic diagram of the piston pump structure.

This study focuses on the swash plate type axial piston pump. The schematic diagram of the piston pump structure is shown in Figure 1. The key components include the drive shaft, bearings, valve plate, cylinder block, pistons, slippers, and swashplate. The piston pump acts as a power unit that converts mechanical energy into hydraulic energy. Mechanical power is transmitted to the cylinder block via shafts. The cylinder block, combined with the piston and sliding shoe assembly, converts oscillating motion into linear reciprocating motion. The reciprocating movement of each piston causes periodic volume changes in its respective piston chamber. This cycle draws in low-pressure fluid through the inlet port and expels high-pressure fluid through the outlet port. Each piston chamber communicates with ports on the valve plate, which are connected to the pump inlet and outlet, facilitating fluid intake and discharge.

**Figure 2:**
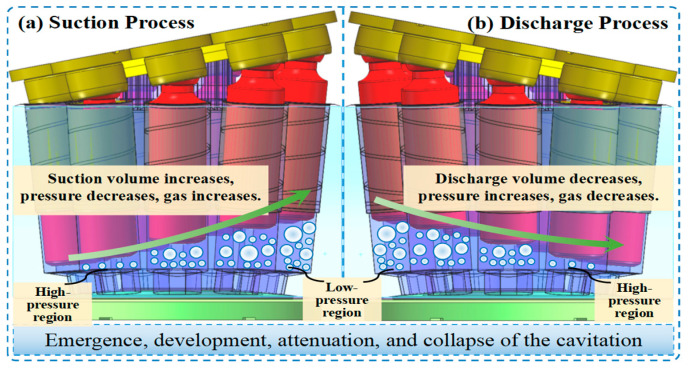
Process of cavitation formation, development, and collapse.

Figure 2 illustrates the complete process of cavitation inception, development, and collapse. During the suction process of the piston pump, the piston moves outward within its cylinder bore. As a result, the sealed volume of the suction chamber increases, creating a localized low-pressure region. If the pressure in this region drops below the saturated vapor pressure of the hydraulic oil, dissolved gases are released, forming bubbles. Simultaneously, partial vaporization of the oil may occur, producing vapor bubbles and initiating cavitation. As the piston moves back inward, the suction chamber volume decreases and the pressure increases. Under this high pressure, the bubbles implode violently, generating localized high-temperature, high-pressure micro-jets that impinge on critical components such as the piston, cylinder bore, and valve plate. This repeated impingement leads to material fatigue and erosion, directly impairing pump performance and service life.

Furthermore, cavitation induces pressure fluctuations in the hydraulic oil, resulting in noise and vibration. These effects accelerate component wear, compromise sealing integrity, and reduce the volumetric efficiency of the pump.

### 2.2. Mathematical Models

The hydraulic oil within the piston pump is a mixture composed of gas and liquid phases, and its behavior is governed by the following equilibrium equation.

(1) $$\frac{\partial \rho_{ad}}{\partial t}+\nabla (\rho_{ad}\mu_{av})=0$$

where $\rho_{ad}$ is the average density of the gas–liquid two-phase flow, $\mu_{av}$ is the average velocity of the gas–liquid two-phase flow.

During operation, the oil in the gas–liquid two-phase flow experiences varying pressures at different locations. The pressure coefficient $C_{p}(x_{i})$ is used to represent the pressure variation at a specific point in the oil.

(2) $$C_{p}=2(p_{i}-p_{\infty })/(\rho_{pump}V_{pump}^{2})$$

where $p_{i}$ is the pressure at a point in the fluid, $p_{\infty }$ is the reference pressure of the fluid, $\rho_{pump}$ is the density of the oil within the piston pump, $V_{pump}$ is the flow velocity of the oil.

When $p_{min}<p_{0}$ in the oil fluid, gas will separate out to form cavitation. Defining $p_{min}=p_{0}$ as the cavitation initiation state, the cavitation initiation coefficient is expressed as

(3) $$\sigma =2(p_{\infty }-p_{0})/(\rho_{pump}V_{pump}^{2})$$

where σ is the cavitation initiation coefficient, $p_{0}$ is the saturated vapor pressure.

Simultaneously, from a microscopic perspective of cavitation, the kinetic equation for bubble formation is introduced to model cavitation. The bubble radius can be described as

(4) $$\frac{dR_{b}}{dt}=\mathrm{sgn}(p_{0}-p_{i})\sqrt{\frac{2(p_{0}-p_{i})}{3\rho_{pump}}}$$

where $R_{b}$ is the radius of the cavitation bubble.

Considering the surface tension coefficient of bubbles, the bubble generation rate and compression ratio can be expressed as

(5) $$R_{gr}=C_{y}(1-f_{e})\frac{\rho_{e}\rho_{pump}}{\sigma_{g}}\sqrt{\frac{2K(p_{0}-p_{i})}{3\rho_{pump}}}$$

(6) $$R_{co}=C_{c}f_{e}\frac{\rho_{e}\rho_{pump}}{\sigma_{g}}\sqrt{\frac{2K(p_{0}-p_{i})}{3\rho_{pump}}}$$

where $R_{gr}$ is the bubble generation rate, $R_{co}$ is the bubble compression ratio, $f_{e}$ is the gas mass fraction, $\sigma_{g}$ is the gas surface tension coefficient, K is the transfer coefficient, $C_{y}$ and $C_{c}$ are constants.

## 3. Simulation Analysis

### 3.1. Simulation Model

**Figure 3:**
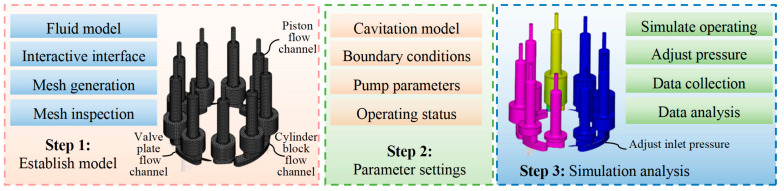
Schematic diagram of simulation analysis.

This study employs the computational fluid dynamics (CFD) method and utilizes the PumpLinx v4.6.0 simulation software to conduct simulation analysis on the system characteristics of piston pumps under different cavitation levels. Figure 3 presents a schematic diagram of the simulation analysis, which primarily consists of three steps. Firstly, the three-dimensional geometric model of the piston pump fluid domain is constructed using SolidWorks 2012 software, imported into the PumpLinx simulation platform, with interfaces constructed to ensure geometric feature integrity, and the fluid domain is discretized using high-precision Cartesian hexahedral meshes to enhance subsequent simulation accuracy. Subsequently, the necessary parameters for the simulation model are set, including the cavitation model, boundary conditions, parameters of the research object, and operating conditions. Finally, different cavitation levels are simulated by adjusting the inlet pressure. Meanwhile, the necessary data from the simulation analysis is collected and analyzed. Table 1 lists the key structural parameters of the piston pump under investigation, which mainly include displacement, rated pressure, peak pressure, maximum speed, number of pistons, and swash plate inclination angle.

### 3.2. Simulation Result

**Figure 4:**
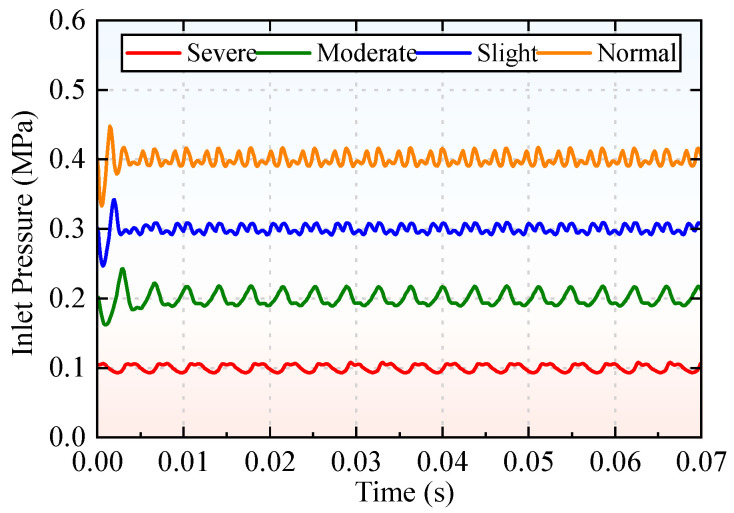
Different cavitation levels corresponding to different inlet pressures.

A significant negative correlation exists between the cavitation severity of the piston pump and its inlet pressure. When the hydraulic oil flows from the oil tank into the piston pump through the suction line, the pressure is highly prone to dropping below the vapor pressure of the oil. Conversely, if the inlet pressure of the piston pump is maintained at a relatively high level and consistently exceeds the oil vapor pressure, the release of dissolved gases from the oil is effectively inhibited, and cavitation can thus be suppressed significantly. Figure 4 illustrates the four cavitation levels corresponding to different inlet pressures of the piston pump. Specifically, by setting the piston pump inlet pressure to 0.4 MPa, 0.3 MPa, 0.2 MPa, and 0.1 MPa, respectively, the corresponding cavitation levels are defined as four states: normal operation, mild cavitation, moderate cavitation, and severe cavitation.

**Figure 5:**
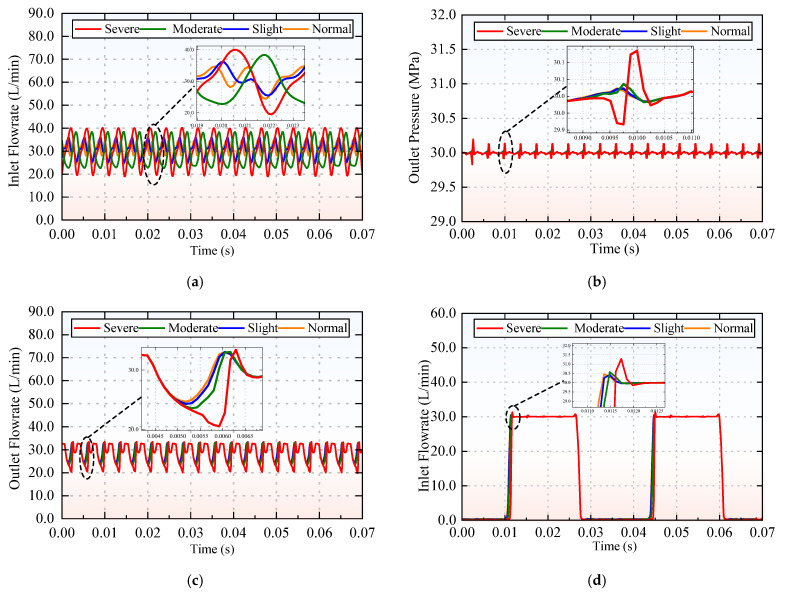
Characteristic curves of the different cavitation levels: (**a**) inlet flow rate; (**b**) outlet pressure; (**c**) outlet flow rate; and (**d**) piston chamber pressure.

Based on the four cavitation states corresponding to different inlet pressures, the pressure and flow characteristics at the key positions of the piston pump are analyzed. Figure 5 presents the characteristic curves of the piston pump under various cavitation levels, including inlet flow rate, outlet pressure, outlet flow rate, and piston chamber pressure. As an axial piston pump is a positive-displacement hydraulic pump, all curves exhibit an overall periodic variation throughout its continuous oil suction and discharge cycles.

In the normal cavitation state, only a small quantity of cavitation bubbles is generated, and these bubbles dissolve in the surrounding hydraulic fluid. With the gradual deterioration of cavitation severity, the number of cavitation bubbles increases progressively—this is accompanied by increasingly significant fluctuations in pressure and flow rate at the key positions of the piston pump. Figure 5a illustrates the inlet flow rate. As shown in Figure 5a,c, the flow fluctuations become more pronounced with intensifying cavitation. Figure 5b shows the outlet pressure. Since the research object is a nine-piston axial piston pump, the pressure presents nine distinct peaks during one full rotation of the cylinder block. Notably, both the pressure and flow rate of the pistons display increasingly large fluctuations with worsening cavitation. Figure 5c depicts the outlet flow rate. Consistent with the structural characteristics of the nine-piston pump, the flow rate also forms nine peaks per complete rotation of the cylinder block. Figure 5d presents the dynamic pressure of the piston chamber. When the piston pump operates in the oil discharge zone, the piston chamber pressure rises to the rated pressure of 30 MPa with a certain degree of fluctuation. When it operates in the oil suction zone, the pressure in the piston chamber drops to near atmospheric pressure. The variation trend of this curve clearly indicates that the piston pump alternates between the oil discharge and suction phases during operation.

## 4. Rough Set Attribute Weighted Convolutional Neural Network

### 4.1. Rough Set Theory

The rough set theory, proposed by Polish mathematician Zdzisław Pawlak, is a mathematical framework for handling incomplete, imprecise, and vague data. Its core idea lies in describing unknown and ambiguous concepts using known and observable information. Without relying on prior knowledge external to the data, it excavates hidden patterns solely through the indiscernibility of data. The fundamental theories of rough sets are defined and described as follows [31,32].

The first definition is the knowledge representation system. ES=(U,A,V,f) knowledge representation system is composed of universe of discourse *U*, attribute set *A*, value domain *V*, and factor mapping relationship *f*. The mapping f:U×A→V assigns values to all attributes within the system. For any a∈A and x∈U, then $f\left(x,a\right)\in V_{a}$. If the *A* satisfies the conditions that A=C∪D and C∩D=∅ where denotes the conditional attribute set *C* and denotes the decision attribute set *D* then the system constituted by and is defined as a decision table [33].

The second definition is the indiscernibility relation. For B⊆A, the indistinguishable relationship *IND*(*B*) is described as follows:

(7) $$IND(B)=\{(x,y)|(x,y)\in U^{2},\forall b\in B(b(x)=b(y))\}$$

The *IND*(*B*) is an equivalence relation, satisfying $IND(B)=\underset{b\in B}{\cap }IND(\{b\})$.

The decision table are ES=(U,A,V,f) and A=C∪D. The breakpoints in the domain $V_{a}$ are denoted as (*a*, *c*). Selecting a set of breakpoints $\left\{\left({{a,c}_{1}}^{a}\right),\left({{a,c}_{2}}^{a}\right),\cdots ,\left({{a,c}_{k_{a}}}^{a}\right)\right\}$ for $\left[h_{a},l_{a}\right]$ within the domain $V_{a}$ defines the classification $P_{a}$.

(8) $$P_{a}=\left\{\left[c_{0}^{a},c_{1}^{a}\right),\left[c_{1}^{a},c_{2}^{a}\right),\cdots ,\left[{c_{ka}}^{a},{c_{ka+1}}^{a}\right]\right\}$$

(9) $$h_{a}=c_{0}^{a}<c_{1}^{a}<c_{2}^{a}<L<{c_{k_{a}}}^{a}<{c_{k_{a}+1}}^{a}=l_{a}$$

(10) $$V_{a}=\left[c_{0}^{a},c_{1}^{a}\right)\cup \left[c_{1}^{a},c_{2}^{a}\right)\cup \cdots \cup \left[{c_{k_{a}}}^{a},{c_{k_{a}+1}}^{a}\right]$$

Arbitrary classification $P=\underset{a\in R}{\cup }P_{a}$ constitutes a new decision-making system.

(11) $$ES=\left(U,A,V^{P},f^{P}\right)$$

(12) $$f^{P}\left(x_{a}\right)=i\Leftrightarrow f\left(x_{a}\right)\in \left[{c_{i}}^{a},{c_{i+1}}^{a}\right)$$

Divide the values of a conditional attribute into equidistant intervals based on specified parameters, disregarding the number of values within each interval. If the maximum and minimum values of a given attribute are $x_{max}$ and $x_{min}$, respectively, and a parameter *k* is specified such that the distance between intervals is $\delta =\left(x_{max}-x_{min}\right)/k$, this can be expressed as $x_{min}+i\delta ,i=0,\ldots ,k$.

Attribute reduction: Ensures diagnostic capability remains unchanged while retaining critical knowledge and eliminating irrelevant information.

Relative reduction: If H⊆J, the *H* is a *K*-reduction in *J*, and only if the *H* is a *K*-independent subfamily of *J* and $pos_{ind(H)}(K)={pos}_{ind(J)}\left(K\right)$. The *K*-reductions in *J* are relative reductions.

In the ES=(U,A,V,f), the importance of each attribute can be computed without relying on prior knowledge based on rough set theory. $Z_{C}(D)$ represents the degree of dependence of attribute *C* on category *D*. When removing a specific attribute from the overall attribute set *C*, the change in $Z_{C}(D)$ can be used to evaluate the attribute importance. The attribute importance $\beta_{D}(C_{i})$ is defined as follows:

(13) $$\beta_{D}(C_{i})=\frac{Z_{C}(D)-Z_{C-C_{i}}(D)}{Z_{C}(D)}=1-\frac{Z_{C-C_{i}}(D)}{Z_{C}(D)}$$

where $Z_{C}(D)$ represents the dependency level of attribute *C* on category *D*, $Z_{C-C_{i}}(D)$ represents the dependency level of category *D* after removing attribute *C*, a smaller $\beta_{D}(C_{i})$ value indicates that the attribute has a lesser impact on the entire system and a smaller influence on fault diagnosis, making it an unimportant attribute. Conversely, a larger $\beta_{D}(C_{i})$ value indicates an important attribute.

The steps for calculating attribute weights primarily consist of the following four parts.

(1) Determine the cavitation failure decision attribute D and the conditional attribute set *C*.
(2) Establish a discretized decision table.
(3) Calculate the values of $Z_{C}(D)$ and $Z_{C-C_{i}}(D)$, respectively, using the following expressions:

(14) $$Z_{C}(D)=\frac{\left|POS_{C}(D)\right|}{\left|U\right|}$$

where $\left|POS_{C}(D)\right|$ denotes the number of positive regions for attribute *C* regarding category *D*, and |U| denotes the number of domains.

(15) $$Z_{C-C_{i}}(D)=\frac{\left|POS_{C-C_{i}}(D)\right|}{\left|U\right|}$$

where $\left|POS_{C-C_{i}}(D)\right|$ denotes the number of positive regions in category *D* after removing attribute *C*.

(4) Finally, calculate the weight for each conditional attribute as described below:

(16) $$\alpha_{i}=\frac{\beta_{D}(C_{i})}{\sum_{i=1}^{n}\beta_{D}(C_{i})}$$

where $\sum_{i=1}^{n}\beta_{D}(C_{i})$ represents the sum of all condition attribute weights.

### 4.2. Convolutional Neural Network

A convolutional neural network (CNN), a typical feedforward neural network, features sparse connectivity and weight sharing. Sparse connectivity, through spatial topological structures, creates a non-fully connected relationship between adjacent layers, reducing the number of parameters for model training. Weight sharing mainly serves to prevent algorithmic overfitting. The CNNs have been widely applied in fields such as image recognition and fault diagnosis [34].

**Figure 6:**
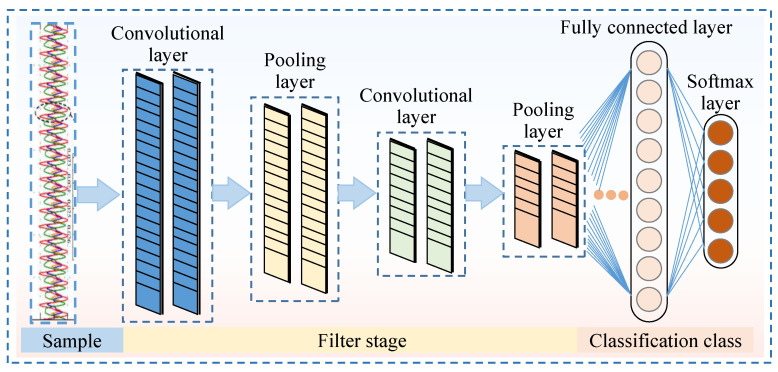
Schematic diagram of the 1D CNN architecture.

Depending on the type of input data, the CNN architectures can be categorized into 1D (one-dimensional), 2D (two-dimensional), and 3D (three-dimensional) models. Since the signals collected in this study, such as pressure and flow rate signals of the piston pump, are data-type signals, the 1D CNN is selected for fault diagnosis, with its network structure illustrated in Figure 6. The 1D CNN architecture comprises an input layer, convolutional layers, pooling layers, fully connected layers, and an output layer. During the early feature extraction phase of the CNN, convolutional layers and pooling layers are alternately utilized to extract features from the input data. Near the output layer, a conventional multi-layer neural network structure is employed.

In the convolutional layer, the convolution kernel performs a convolution operation on the feature vectors output by the previous layer. A nonlinear activation function is then used to construct the output feature vectors. The output of each convolutional layer corresponds to the convolution results of multiple input features, and its mathematical model can be described as follows:

(17) $$x_{j}^{\left(l\right)}=f\left(\sum_{d}k_{j,d}^{\left(l\right)}*x_{d}^{\left(l-1\right)}+b_{j}^{\left(l\right)}\right)$$

where *l* denotes the number of network layers, *j* represents the number of channels, *f* is the activation function, $k_{j,d}^{\left(l\right)}$ is the *d*th vector in the *j*th convolution kernel, and $b_{j}^{\left(l\right)}$ is the network bias term.

The pooling layer maps data from the convolutional layer, typically employing a max pooling operator. This operator extracts local maxima from data features, thereby reducing the number of training parameters while enhancing feature robustness. The max pooling function can be described as

(18) $$T_{j}^{\left(l+1\right)}=\underset{(j-1)W+1\leq t\leq jW}{max}\left\{m_{j}^{l}(t)\right\}$$

where $m_{j}^{l}(t)$ is the *j*th feature vector in the *l*th pooling layer, *t* ranges over $\left[(j-1)W+1,jW\right]$, *W* is the pooling width, $T_{j}^{\left(l+1\right)}$ is the value of the *l* + 1th layer neuron.

The output of the 1D CNN employs the Softmax classifier to address the fault classification problem. The function can be expressed as

(19) $$O=f(b_{o}+w_{o}f_{v})$$

where $b_{o}$ and $w_{o}$ represent the bias vector and weight matrix, respectively, and $f_{v}$ denotes the feature vector.

## 5. Fault Diagnosis Process and Results

### 5.1. Fault Diagnosis Model

**Figure 7:**
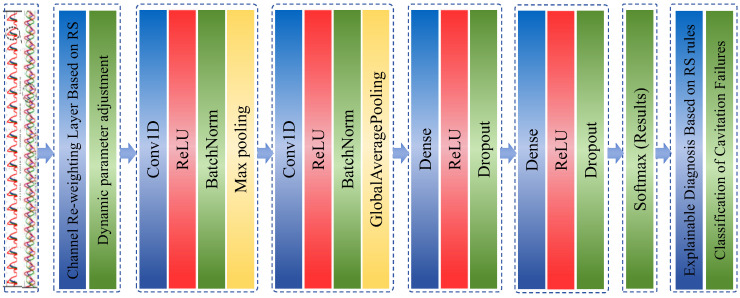
Flowchart of the RSAW-CNN-based cavitation fault diagnosis model.

This paper puts forward an intelligent fault diagnosis method based on the rough set attribute weighted convolutional neural network (RSAW-CNN). As shown in Figure 7, the workflow and architecture of this state recognition method are presented. The core architecture consists of the following components: data input layer, rough-set (RS)-based attribute weight assignment layer, convolutional layers, pooling layers, fully connected layers, result output layer, and RS-rule-based auxiliary decision-making layer.

(1) Data input layer.

A transient computational fluid dynamics (CFD) model of the axial piston pump is established. This CFD model is used to generate signals (e.g., flow rate and pressure) for the healthy axial piston pump; meanwhile, based on the cavitation mechanism of the piston pump, it simulates flow rate, pressure, and other signals under different cavitation levels. Both the healthy and cavitation fault signals are collected as the original dataset for subsequent processing.

(2) Rough set attribute weighted layer.

The input channel signals are preprocessed to identify condition attributes and decision attributes for plunger pump cavitation faults based on rough set theory, thereby establishing the original fault decision table. The table is then discretized to obtain a discretized decision table. Attribute dependencies are calculated to determine channel weights, which are dynamically adjusted to highlight key channel information most relevant to cavitation faults. This process not only optimizes feature extraction but also significantly enhances the interpretability of the network model.

(3) Convolutional layers.

The convolutional layer module consists of two one-dimensional convolutional layers designed to extract local features from the input signal. The ReLU activation function is then applied to provide nonlinear transformation capabilities, enabling the model to learn more complex feature relationships. Concurrently, data normalization is employed to accelerate training convergence and improve the model’s overall stability.

(4) Pooling layers.

Max pooling and global average pooling are implemented. These operations reduce the number of parameters while preserving the most significant features, thereby enhancing the model’s anti-interference ability.

(5) Fully connected layers.

Two fully connected layers are employed. The first layer integrates the extracted local features into a global representation, while the second layer functions as the classifier for cavitation faults. A Dropout layer is also incorporated to further prevent overfitting.

(6) Result output layer and RS rule-based auxiliary decision-making layer.

This layer outputs the classification results for cavitation faults. Based on rough set theory, we first construct the initial decision table for piston pump cavitation faults. Then, through data discretization, we obtain a discrete decision table. Next, we apply genetic algorithms for attribute reduction, extracting the minimal reduced attribute set and its corresponding decision rules. These rules are used to assist in cavitation fault diagnosis, effectively enhancing the stability, interpretability, and credibility of the RSAW-CNN model. This approach meets the industrial demand for trustworthy artificial intelligence models.

Table 2 presents the parameters of the fault diagnosis model. The configured model parameters primarily include input shape, rough set attribute weighted layer, convolution layer, pooling layer, dense layer, Softmax layer, and optimizer.

### 5.2. Fault Diagnosis Process

The piston pump operates at an outlet pressure of 30 MPa and a rotational speed of 1800 r/min. Cavitation of varying degrees is induced by adjusting the inlet pressure of the piston pump. Specifically, inlet pressures of 0.4 MPa, 0.3 MPa, 0.2 MPa, and 0.1 MPa are set, which represent four cavitation levels: normal, no cavitation, mild cavitation, and severe cavitation. A total of 4800 datasets are collected across the four cavitation levels (one set corresponding to a 5 s continuous operation cycle of the piston pump), with 1200 sets per level to ensure balanced sample distribution. Among these, training samples constitute 80% of the total (3840 sets) for model training and parameter optimization, while test samples comprise the remaining 20% (960 sets) for independent validation of diagnostic performance, as shown in Table 3.

This paper applies wavelet transform noise filtering to the acquired signals. The dbN wavelet family is selected for signal decomposition and reconstruction. The signal undergoes five-level decomposition and reconstruction using wavelet basis functions. The noise-reduced signals achieve a signal-to-noise ratio exceeding 30 dB, demonstrating effective noise reduction.

Computing attribute weights and extracting rules based on rough set theory. Features are extracted based on the rough set theory. The domain U represents 4800 sample datasets. The conditional attributes C_1_–C_5_ are outlet pressure, outlet flow rate, inlet flow rate, piston chamber pressure, and piston chamber volume. The decision attribute D is categorized as normal, slight cavitation, moderate cavitation, and severe cavitation, labeled as classes 0, 1, 2, and 3, respectively. This constructs the decision table for cavitation faults, as shown in Table 4.

The core of rough set theory lies in indistinguishable relations and equivalence classes, requiring the discretization of continuous attributes. Signals such as the outlet pressure of the piston pump are continuous and must be discretized. After equidistant discretization of the decision table for piston pump cavitation faults, the resulting discrete cavitation table is shown in Table 5.

The attribute importance and weights for the five input channels (conditional attributes) are calculated according to Equation (16), as shown in Table 6.

Compared to classical attribute reduction algorithms, the genetic algorithm is more suitable for handling large-scale and high-dimensional data, and it is less prone to becoming trapped in local optima, thereby having a higher probability of finding the global optimal reduction subset. Using attribute reduction based on the genetic algorithm, five reduced conditional attributes are obtained: outlet pressure, outlet flow rate, inlet flow rate, piston chamber pressure, and piston chamber volume. We have explicitly set the key parameters of the genetic algorithm. The population size is 335; the maximum number of iterations is 200; the crossover method adopts single-point crossover with a crossover probability of 0.8; the mutation method uses bit-flipping mutation with a mutation probability of 0.01. Meanwhile, we have clarified the core mechanism of the algorithm: each individual is encoded as a binary string, whose length is equal to the number of original conditional attributes. A bit value of 1 indicates that the corresponding attribute is selected into the reduced subset, while 0 indicates elimination. The fitness function is defined as Fitness = 0.9 × classification accuracy + 0.1 × (1 − reduction rate). This function aims to balance classification accuracy and the parsimony of the attribute set, where classification accuracy refers to the accuracy of the decision table composed of the subset obtained through rough set classification, and reduction rate is the ratio of the size of the reduced subset to the size of the original attribute set. The weight coefficient of 0.9 implies that we prioritize maintaining high classification performance. Retaining the reduced attributes and removing duplicate rows yields the minimal decision table presented in Table 7.

The decision rules derived from rough sets are shown in Table 8. Each rule consists of conditional attributes (different monitoring features) and a decision attribute (cavitation fault type or severity), forming a conditional logic. For example, Rule 1 can be interpreted as follows: If the outlet pressure is in the range of 29.9701 MPa to 30.0237 MPa, the outlet flow rate is in 30.4636 mL/r to 34.3511 mL/r, the inlet flow rate is in 24.2675 mL/r to 29.7890 mL/r, the piston chamber pressure is in 0.0576 MPa to 7.7585 MPa, and the piston chamber volume is in 0.0019 L to 0.0024 L, then the decision result is normal. The Right-Hand Side Coverage is 0.016667, and the RHS Accuracy is 0.454545. These rules transform the implicit features of piston pump cavitation faults into interpretable quantitative interval logic. In practical decision-making support, they not only provide a method to quickly determine fault types based on parameter intervals but also quantify the reliability of each rule through coverage and accuracy indicators.

**Figure 8:**
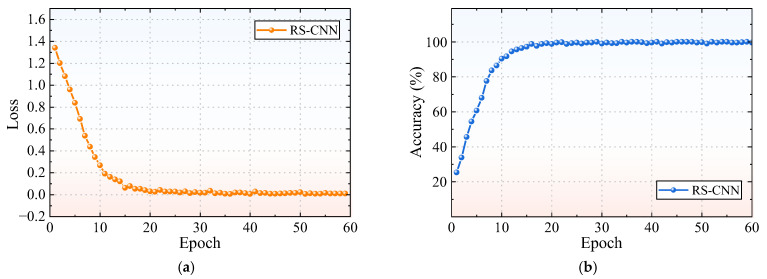
Experimental results of the RSAW-CNN: (**a**) Loss; (**b**) Accuracy.

To mitigate the effects of random variations, ten trials are conducted to improve the reliability and accuracy of the results. The loss rate and accuracy of the RSAW-CNN diagnostic method are presented in Figure 8.

In Figure 8a, as the number of epochs increases, the loss rate gradually decreases and tends to be stable and finally stabilizes close to 0. This indicates that the prediction error of the model for the fault diagnosis task is continuously reduced, and the learning effect is good. From Figure 8b, it can be seen that the accuracy of the RSAW-CNN model rises rapidly with the increase in epochs. After the number of epochs reaches a certain value (about 22), the accuracy gradually becomes stable and finally stabilizes at a high level close to 100%. This shows that the model can perform fault diagnosis very accurately and has high classification performance.

### 5.3. Comparative Analysis

To validate the effectiveness of the proposed RSAW-CNN model, a comparative analysis has been carried out with five diagnostic approaches: Backpropagation Neural Network (BPNN), Residual Neural Network (ResNet), conventional Convolutional Neural Network (CNN), CNN-SENet (a CNN incorporating a squeeze-and-excitation attention mechanism) [35], and the proposed RSAW-CNN model. To maintain consistency, the CNN architecture and input data remain identical for all models. The input data comprises five-channel cavitation fault signals collected from a piston pump, with 3840 samples used for training and 960 for testing. Each experiment has been repeated ten times, and the average of the results is considered as the final outcome.

**Figure 9:**
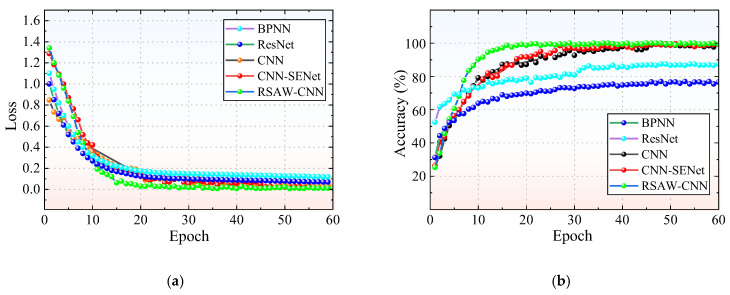
Comparison of test results for different diagnostic methods: (**a**) Loss; (**b**) Accuracy.

Figure 9 illustrates the loss and accuracy curves of the five diagnostic methods. The loss values of all methods eventually approach zero, which indicates no overfitting occurs. Among them, the RSAW-CNN model demonstrates the best performance, showing higher stability and superior diagnostic accuracy compared to the other four methods. In the later training stages, the loss of the proposed RSAW-CNN model converges to a value close to zero (0.006). The accuracy reaches 100% by the 22nd epoch and remains stable afterwards. The CNN-SENet model exhibits the second-best performance, while the baseline CNN outperforms ResNet. The BPNN model displays the lowest performance among all the methods compared.

**Figure 10:**
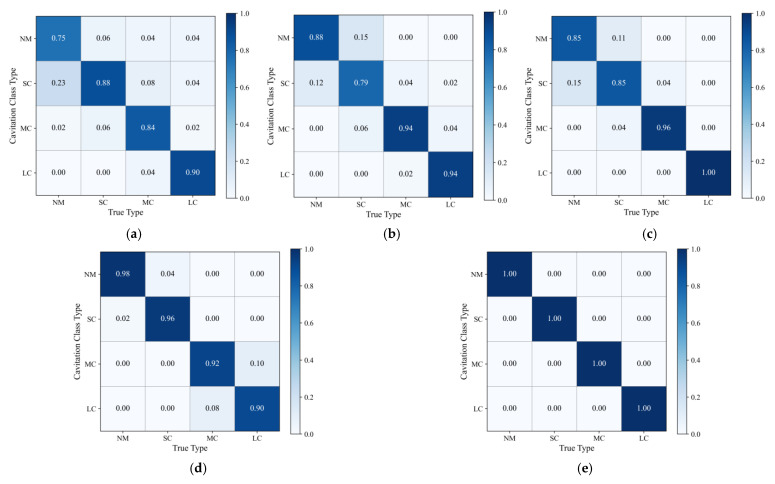
Confusion matrix for five fault diagnosis methods: (**a**) BPNN; (**b**) ResNet; (**c**) CNN; (**d**) CNN-SENet; (**e**) RSAW-CNN.

As shown in Figure 10, Figure 10a–e illustrate the diagnostic performance of five methods (BPNN, ResNet, CNN, CNN-SENet, and RSAW-CNN) for cavitation faults via confusion matrices. The vertical axis displays the model’s diagnostic results, while the horizontal axis shows the actual fault labels. The labels NM, SC, MC, and LC denote the following: NM for Normal Condition, SC for Slight Cavitation, MC for Moderate Cavitation, and LC for Severe Cavitation. The BPNN model struggles to accurately distinguish between Normal Condition and Slight Cavitation, as well as between Slight Cavitation and Moderate Cavitation. The ResNet and CNN models also fail to accurately differentiate between Normal Condition and Slight Cavitation, while CNN-SENet cannot precisely identify Moderate and Severe Cavitation. The primary reasons are as follows: the BPNN model is prone to local optimization issues; the ResNet model suffers from low computational efficiency due to its complex parameters; and the pure CNN treats the importance of each channel as equivalent, failing to capture subtle differences in weak faults, thereby generating diagnostic errors and resulting in inconsistent fault identification capabilities. As shown in Figure 10e, compared to the other four methods, RSAW-CNN can accurately identify cavitation faults across all categories. The model demonstrates robust performance and high diagnostic accuracy, making it well-suited for piston pump cavitation fault diagnosis.

**Figure 11:**
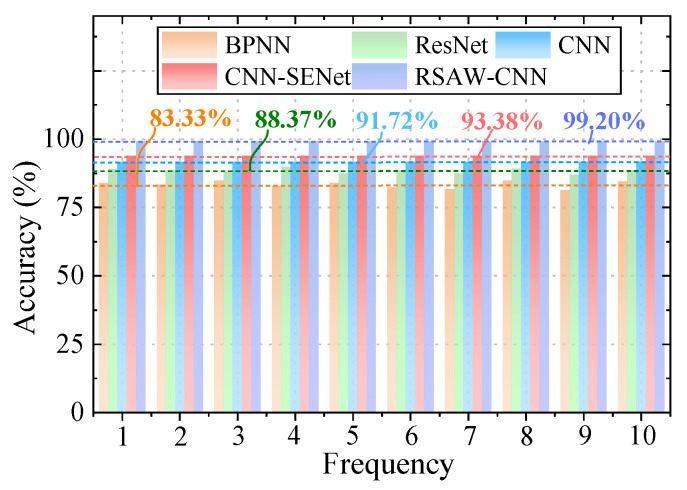
Average accuracy of three methods.

The proposed RSAW-CNN method employs a rough set theory-weighted convolutional neural network diagnostic approach. It focuses more on critical information across multiple channels, allocates weights rationally, and utilizes rough set decision rules to assist diagnosis. This enables accurate identification of cavitation faults across various categories, enhancing the interpretability and reliability of fault diagnosis. Figure 11 shows the average accuracy across ten trials on the dataset. The BPNN method achieves an average accuracy of 83.33%, the ResNet method reaches 88.37%, the CNN method attains 91.72%, and the CNN-SENet method achieves 93.80%. Comparative analysis with other methods demonstrates that the RSAW-CNN method exhibits significant advantages in performance metrics such as accuracy and training speed, with an average accuracy of 99.2%. Compared to the BPNN, ResNet, CNN, and CNN-SENet methods, its average accuracy has improved by 15.87%, 10.83%, 7.48%, and 5.40%, respectively. This validates the proposed method’s significant advantages in diagnosing cavitation faults in piston pumps.

## 6. Conclusions

The proposed rough set attribute weighted convolutional neural network (RSAW-CNN) model does not rely on prior knowledge. It assigns weights to each channel sensor based on conditional attribute weights, enabling the model to focus on important conditional attributes and enhance classification performance. The model learns classification rules from data, embodying them as weights, thereby improving model interpretability and credibility. Through multiple experiments, the RSAW-CNN fault diagnosis model demonstrated high accuracy for piston pump cavitation diagnosis, achieving an average accuracy of 99.2% with stable convergence. Compared to the BPNN, ResNet, CNN, and CNN-SENet, its average accuracy has improved by 15.87%, 10.83%, 7.48%, and 5.40%, exhibiting higher accuracy and greater stability.

The RSAW-CNN fault diagnosis method employs rough set theory to extract rules for auxiliary diagnosis. While ensuring high accuracy, it significantly enhances the reliability and credibility of the diagnostic process, aligning with industry demands for trustworthy AI. This fully demonstrates the method’s application value in future industrial equipment health management, driving axial piston pump fault diagnosis toward smarter, more reliable, and more efficient solutions.

## Figures and Tables

**Table 1:** Main parameters of the piston pump.

Number	Parameter	Value	Unit
1	Pump displacement	18	mL/r
2	Rated pressure	30	MPa
3	Peak pressure	32	MPa
4	Max. rotation speed	3900	rpm
5	Piston number	9	/
6	Swash plate inclination	7	°

**Table 2:** Parameters of the RSAW-CNN-based cavitation fault diagnosis model.

Model	1D CNN
Input shape	(4800, 5)
Rough set attribute weighted layer	Initial weight = [0.343707, 0.339648, 0.125846, 0.096075, 0.094724]
Convolution layer 1	Filters = 16, Kernel size = 3, Activation = ‘ReLU’
Max pooling layer	Pool size = 2
Convolution layer 2	Filters = 32, Kernel size = 3, Activation = ‘ReLU’
Global average pooling	Pool size = 2
Dense layer 1	Dropout (0.3) + ReLU
Dense layer 2	Dropout (0.2) + ReLU
Softmax layer	Classification of Cavitation Failures
Optimizer	Adam

**Table 3:** Four cavitation levels and fault data.

Inlet Pressure (MPa)	Cavitation Rate (%)	Cavitation Severity	Total Samples	Training Samples	Test Samples
0.4 MPa	0	Normal	1200	960	240
0.3 MPa	1.0%	Mild	1200	960	240
0.2 MPa	2.0%	Moderate	1200	960	240
0.1 MPa	8.0%	Severe	1200	960	240

**Table 4:** Decision table for cavitation failures.

Samples	C_1_	C_2_	C_3_	C_4_	C_5_	D
1	30.000486	32.717197	28.460391	0.194822	0.001425	0
2	29.998681	32.825382	29.805336	0.193485	0.001429	0
…	…	…	…	…	…	…
601	29.997748	32.008094	29.603951	0.098347	0.001444	1
602	29.995564	31.560013	30.328955	0.080565	0.001451	1
…	…	…	…	…	…	…
4800	30.002306	30.606237	38.107013	0.042861	0.002342	3

**Table 5:** Discrete cavitation table for piston pump.

Samples	C_1_	C_2_	C_3_	C_4_	C_5_	D
1	1	3	1	0	0	0
2	1	3	2	0	0	0
…	…	…	…	…	…	…
601	1	3	1	0	0	1
602	1	3	1	0	0	1
…	…	…	…	…	…	…
4800	1	3	3	0	1	3

**Table 6:** Rough set attribute weight table.

Condition Attribute	Attribute Importance	Weight
Condition attribute 1	0.105833	0.343707
Condition attribute 2	0.104583	0.339648
Condition attribute 3	0.038750	0.125846
Condition attribute 4	0.029583	0.096075
Condition attribute 5	0.029167	0.094724

**Table 7:** Minimal decision table.

Samples	C_1_	C_2_	C_3_	C_4_	C_5_	D
1	1	3	1	0	0	0
2	1	3	2	0	0	0
…	…	…	…	…	…	…
65	1	3	1	0	0	1
66	1	3	2	0	0	1
…	…	…	…	…	…	…
335	1	2	1	0	1	3

**Table 8:** Decision rules.

Number	Rule	Right-Hand Side Coverage	Right-Hand Side Accuracy
1	If C_1_ ranges from 29.9701 to 30.0237, C_2_ ranges from 30.4636 to 34.3511, C_3_ ranges from 24.2675 to 29.7890, C_4_ ranges from 0.0576 to 7.7585 and C_5_ ranges from 0.0019 to 0.0024, then fault(0)	0.016667	0.454545
2	If C_1_ ranges from 29.9701 to 30.0237, C_2_ ranges from 30.4636 to 34.3511, C_3_ ranges from 24.2675 to 29.7890, C_4_ ranges from 0.0576 to 7.7585 and C_5_ ranges from 0.0019 to 0.0024, then fault(1).	0.02	0.545455
3	If C_1_ ranges from 29.9701 to 30.0237, C_2_ ranges from 30.4636 to 34.3511 C_3_ ranges from 18.7459 to 24.2675, C_4_ ranges from 0.0001 to 7.7585 and C_5_ ranges from 0.0019 to 0.0024, then fault(2).	0.011667	1.0
…	…	…	
45	If C_1_ ranges from 29.9701 to 30.0237, C_2_ ranges from 18.8012 to 22.6887, C_3_ ranges from 18.7459 to 24.2675, C_4_ ranges from 23.3907 to 31.2067 and C_5_ ranges from 0.0014 to 0.0019, then fault(3).	0.01	1.0

## Data Availability

The original contributions presented in the study are included in the article, further inquiries can be directed to the corresponding author.

## Abbreviations

The following abbreviations are used in this manuscript:

CFD	Computational Fluid Dynamics
CNN	Convolutional Neural Networks
1D CNN	One-dimensional Convolutional Neural Networks
CNN-SENet	Convolutional Neural Networks Squeeze-and-Excitation Networks
LC	Severe Cavitation
MC	Moderate Cavitation
NM	Normal State
RSAW-CNN	Rough Set Attribute Weighted Convolutional Neural Network
SC	Slight Cavitation
